# Triatomine vectors of *Trypanosoma cruzi* in an endemic area for Chagas disease in Northeast Brazil

**DOI:** 10.1590/0037-8682-0413-2024

**Published:** 2024-02-05

**Authors:** José Atanásio de Oliveira, Daniela Bandeira Anastácio, Tatiene Rossana Móta Silva, Samuel Souza Silva, Gílcia Aparecida de Carvalho, Rafael Antonio Nascimento Ramos

**Affiliations:** 1 Universidade Federal do Agreste de Pernambuco, Laboratório de Parasitologia, Garanhuns, PE, Brasil.; 2 Universidade Federal Rural de Pernambuco, Programa de Pós-Graduação em Biociência Animal, Recife, PE, Brasil.; 3 II Macrorregional de Saúde, Secretaria Estadual de Saúde, Caruaru, PE, Brasil.

**Keywords:** Triatominae, Panstrongylus, Triatoma, Infection rate, Spatial distribution, Trypanosomatidae

## Abstract

**Background:**

We assessed the distribution of triatomines in an endemic area for Chagas disease.

**Methods:**

This retrospective study used secondary data extracted from the Official System of the National Chagas Disease Control Program (*Sistema Oficial do Programa Nacional de Controle da Doença de Chagas* - *SisPCDCh*).

**Results:**

A total of 7,257 (725.7 ± 221.7 per year) specimens were collected from 2013 to 2022. Most of them (6,792; 93.6%) were collected in the intradomicile and 465 (6.4%) in the peridomicile. A total of 513 (7.1%) triatomines tested positive for the presence of trypomastigote forms, similar to *Trypanosoma cruzi*.

**Conclusions:**

The spatial analysis revealed a heterogeneous distribution of triatomines across different municipalities.

Triatomines (Reduviidae: Triatominae) are arthropod vectors of the protozoon *Trypanosoma cruzi* (Kinetoplastida: Trypanosomatidae), which is the causative agent of Chagas disease (CD)^1^. CD is considered a neglected illness of great importance in Latin America. However, epidemiological changes, mainly due to population mobility, urbanization, and emigration, have increased cases in Canada, the United States, European and African countries, the Eastern Mediterranean, and the Western Pacific^2^. It is estimated that globally, approximately 75 million people live in at-risk areas and 6-7 million are infected, with approximately 14,000 deaths annually. In humans, the classical transmission route is through contact with trypomastigote forms of *T. cruzi* shed on the feces of vectors after a blood meal². 

In some Brazilian regions, precarious human dwellings are still observed, which facilitate the establishment and reproduction of triatomine species, because they may serve as shelters. Over the last few years, extensive chemical control of triatomines in households has led to the absence of *T. cruzi* transmission by *Triatoma infestans* in Brazil^3^. However, other species (e.g., *Panstrongylus lutzi*, *Panstrongylus megistus*, *Triatoma brasiliensis*, and *Triatoma pseudomaculata*) continue to play an essential role in *T. cruzi* transmission^4^ ^,^ ^5^. Most present an invasive behavior already found in domestic and peridomestic environments in regions endemic for CD^6^ ^,^ ^7^. 

An important strategy for the Chagas Disease Control Program (ChDCP) is establishing triatomine information posts (PITs). In these places, residents from endemic areas may deliver insects suspected of being kissing bugs for identification by the health surveillance service^8^. Passive surveillance has been an important measure for monitoring the triatomine population and is useful for driving strategies for CD control. A previous study conducted in the state of Pernambuco demonstrated that 8.8% of triatomines collected were naturally infected with *T. cruzi*, and of 10 different species collected, *T. pseudomaculata*, *T. brasiliensis*, and *P. lutzi* predominated^9^.

In times of rapid environmental change and marked anthropogenic influence on the degradation of natural habitats of animals and vectors, knowledge of the distribution of triatomines is pivotal for driving preventive measures against CD^10^ ^,^ ^11^. Therefore, we assessed the distribution and infection rates of *T. cruzi* in triatomines collected from a region endemic for CD in Northeast Brazil.

This retrospective study used secondary data (2013-2022) obtained from the Official System of the National Chagas Disease Control Program (*Sistema Oficial do Programa Nacional de Controle da Doença de Chagas* - *SisPCDCh*) in the state of Pernambuco (Consent Letter 001/2022). Triatomines were actively (Caruaru and Santa Cruz do Capibaribe) and passively obtained from the municipalities and delivered to the *IV Gerência Regional de Saúde do Estado de Pernambuco (IV GERES)*. The database was fed daily at the municipal level and information was sent weekly to the *IV GERES*. The study area comprised 32 municipalities in the Agreste region located in the state of Pernambuco (Figure 1). 

**FIGURE 1:**
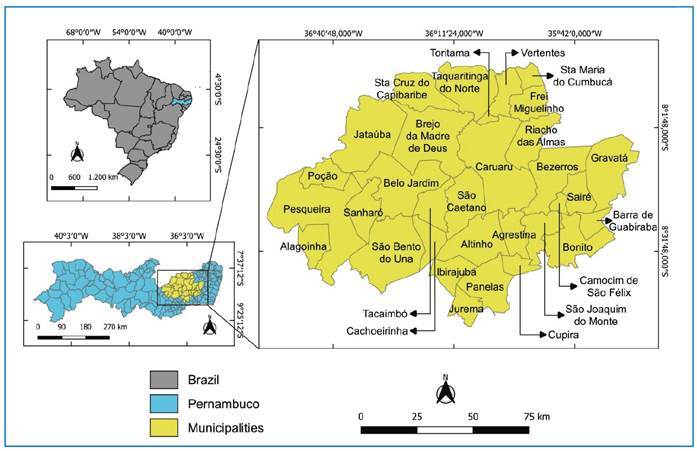
Study area - Municipalities located in the Agreste region of the state of Pernambuco, Brazil.

Data on triatomine species, capture area (intradomicile or peridomicile), and life stage (adult or nymph) were obtained (Supplementary Table 1). According to ChDCP, *T. cruzi* infection was detected by abdominal compression of each specimen followed by microscopic analysis. The abdominal content was placed on a microscope slide with 20 microliters of 0.9% saline solution, covered with a coverslip and analyzed at different magnifications (40× and 100×). 

Infection rates were determined based on absolute and relative frequencies. The chi-square test (χ²) was used to analyze the stages of development of triatomines and ecotypes (intradomicile or peridomicile) and between infected species of triatomines in the ecotypes. The G-test was used to analyze the number of triatomines captured in the intradomicile or peridomicile, the predominant species in these municipalities, and the species in the ecotypes (intradomicile or peridomicile). The significance level was set at 5%, and all analyses were performed using the BioEstat software version 5.3¹².

Maps were produced using QGIS 3.22.10, in which vector layers were inserted into a shapefile obtained from the *Instituto Brasileiro de Geografia e Estatística (IBGE)* database. A thematic map showed the number of triatomines captured in each municipality. A second was represented by the main species captured, and the frequency was represented by a histogram grouped by the color scale.

A total of 7,257 (725.7 ± 221.7 per year) triatomines belonging to nine species were collected during the study period. Most specimens were collected in the intradomicile (6,792; 93.6%) and some (465; 6.4%) in the peridomicile area. Triatomines were collected per year as follows: 2013 (n = 911; 12.6%); 2014 (n = 735; 10.1%); 2015 (n = 1,023; 15%); 2016 (n = 815 11.2%); 2017 (n = 818 11.3%); 2018 (n = 835; 11.5%); 2019 (n = 824; 11.3%); 2020 (n = 550; 7.6%); 2021 (n = 403; 5.5%), and 2022 (n = 343; 4.8%). Caruaru (n = 2,274; 31.3%) was the municipality that registered the highest number of specimens, followed by Santa Cruz do Capibaribe (n = 1,961; 27%) and São Bento do Una (n = 686; 9.4%) (G-test = 232.5792; p = 0.0000).

A total of 6,530 (96.2%) adults and 262 (3.8%) nymphs were collected in the intradomicile, while 337 (72.5%) adults and 128 (27.5%) nymphs were found in peridomicile areas (χ² = 474.815; p = 0.0000). The most common triatomine was *P. lutzi* (n = 2,891; 39.8%), followed by *T. brasiliensis* (n = 2,617; 37%), *T. pseudomaculata* (n = 1,343; 18.5%), and *P. megistus* (n = 314; 4.32%) (G-test = 86.9616; p = 0.0000). Data for all collected triatomine species and rates of infection by *T. cruzi* are reported in Table 1.

Out of 7,240 triatomines tested, 7.1% (n = 513) were positive for the presence of trypomastigote forms similar to *T. cruzi* (Table 1). The highest number of infected triatomines (n = 114; 12.7%) was recorded in 2013. *Triatoma brasiliensis* (12.2%; 318/2,607), *Triatoma melanocephala* (10%; 1/10), and *P. lutzi* (6.1%; 176/2,890) presented higher rates of infection (Table 1). Some of the collected specimens were not examined (n = 17) because of inadequate sample conditions.

**TABLE 1:** Triatomine species collected and examined for positivity, and infection rate by *T. cruzi*.

Species	Collected			Examined			Positive			Infection rate (%)
	Intra	Peri	Total	Intra	Peri	Total	Intra	Peri	Total	
*P. geniculatus*	2	0	2	2	0	2	0	0	0	0
*P. lutzi*	2,803	88	2,891	2,802	88	2,890	157	19	176	6.1
*P. megistus*	266	48	314	266	47	313	4	0	4	1.3
*R. nasutus*	2	0	2	2	0	2	0	0	0	0
*R. neglectus*	3	0	3	3	0	3	0	0	0	0
*Rhodnius* spp.^1^	19	1	20	19	1	20	0	0	0	0
*T. brasiliensis*	2,427	190	2,617	2,417	190	2,607	293	25	318	12.2
*Triatoma* spp.^1^	1	1	2	1	1	2	0	0	0	0
*T. melanocephala*	9	1	10	9	1	10	1	0	1	10
*T. petrochii*	52	1	53	51	1	52	2	0	2	3.8
*T. pseudomaculata*	1,208	135	1,343	1,205	134	1,339	10	2	12	0.9
Total	6,792	465	7,257	6,777	463	7,240	467	46	513	7.1

**Intra:** intradomicile; **Peri** peridomicile; ^1^Specimens were not identified at species level because of the quality of the sample.

Spatial analysis revealed a heterogeneous distribution of triatomines in the study area. Of the 32 municipalities, 17 had frequencies below 50, whereas Caruaru and Santa Cruz do Capibaribe exhibited the highest frequencies of collected specimens (>1,000) (Figure 2). *Panstrongylus lutzi* and *T. brasiliensis* were predominant in Caruaru, Santa Cruz do Capibaribe, Brejo da Madre de Deus, and Belo Jardim (>50 specimens). For *T. pseudomaculata* and *P. megistus*, a homogeneous distribution was observed among municipalities (<50 specimens) (Figure 3).

**FIGURE 2:**
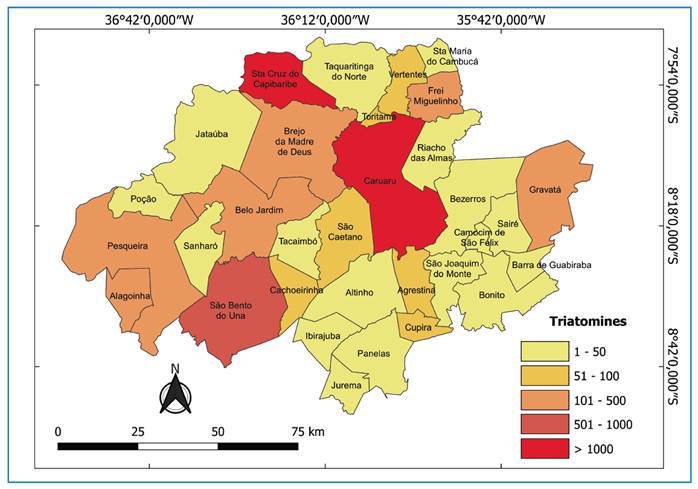
Overall distribution of triatomines collected from 2013 to 2022.

**FIGURE 3:**
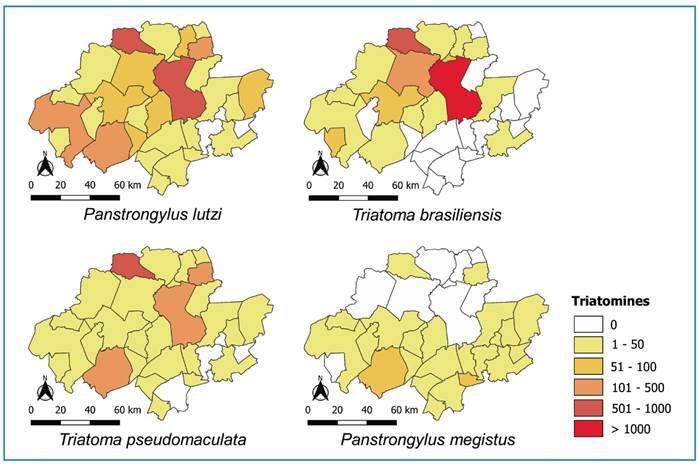
Distribution of *Panstrongylus lutzi*, *Panstrongylus megistus*, *Triatoma brasiliensis*, and *Triatoma pseudomaculata* collected from 2013 to 2022.

This study revealed a wide distribution of triatomine vectors in an endemic area for CD, with a positivity rate of 7.1% for trypanosomatid forms, similar to *T. cruzi*. The collection of most triatomines in the intradomicile has been reported in previous studies, demonstrating their closer proximity to humans and facilitating *T. cruzi* infection on an endemic scale^9^ ^,^ ^13^. Currently, in most municipalities, these specimens are obtained mainly through passive surveillance, in which people collect and deliver them to each municipality's health services. This passive surveillance is likely to be a critical bias influencing the higher number of specimens collected in the intradomicile. The finding of nymphs indoors suggests adaptation and the potential ability to colonize human dwellings, posing an additional risk for human vector exposure and, consequently, *T. cruzi* infection^5^ ^,^ ^14^.

The rising retrieval of triatomines in peri- or intradomicile areas observed across different studies may be a consequence of two main factors: i) climate change, deforestation, and expansion of agricultural areas moving triatomine populations from the original habitat to peri- or intradomicile areas^14^, and ii) the ability of dispersion by flying guided by artificial lights, thus favoring the invasion of houses.

*P. lutzi* likely has great epidemiological importance in the study area since it was reported in almost all municipalities (90.6%). Its absence in three municipalities (Barra de Guabiraba, Camocim de São Félix, and São Joaquim do Monte) may be related to its location in high-altitude areas (from 462 to 723 m above sea level), milder climate, and higher rates of rainfall. In addition, these municipalities are located in an area characterized by typical vegetation of the Atlantic Forest, which differs from the other assessed municipalities. The importance of this vector cannot be underestimated because its natural infection rate and adaptation to domiciles are high. 

Currently, *T. brasiliensis* is considered the primary vector in Northeast Brazil^9^. In Pernambuco, its distribution was previously assessed and found to be predominant over other triatomine species^9^. Despite being more abundant, the infection rate of *T. cruzi* was lower than that of *P. lutzi*. In the present study, many *T. brasiliensis* were collected from the peridomicile, and livestock animals were observed close to the houses. The presence of animals (e.g., cattle, sheep, and goats) close to human dwellings facilitates the establishment of triatomine populations because they have a continuous feeding source^14^.

*Triatoma pseudomaculata* is distributed in the Northeast, Midwest, and Minas Gerais, and can be found mainly in the wild (tree bark, rodents, and marsupial refuges), peridomestic (corrals and chicken coops), and occasionally intradomicile environments^15^. The low infection rate of *T. pseudomaculata* in this study corroborates previous research. Triatomine is a low-efficiency vector with a small rate of infection^15^. 

Despite this, the overall reduction in specimens collected over 10 years could be a consequence of improvements in human dwellings as well as the hygiene conditions of the population. However, it is interesting to note that the health decentralization process that occurred in Brazil in 1999 created important gaps in the control of endemic diseases. The lack of financial resources in municipalities, along with the scarcity of qualified human resources, may also contribute to the reduction in entomological surveillance. 

From 2021 to 2022, the marked reduction in surveillance actions can also be explained by the Coronavirus pandemic; a mean of 336 specimens were collected annually in the study area during the 2 years cited above. In addition, the actions of the ChDCP have been executed unevenly by the municipality’s health services. For instance, in the municipalities of Caruaru and Santa Cruz do Capibaribe, there is active vigilance from a team of professionals exclusively involved in entomological surveillance. In municipalities with active vigilance, there is training of human resources involved in the parasitological examination of triatomines, which can contribute to the positivity observed in these vectors. Improving entomological surveillance (e.g., active surveillance, chemical control, and educational programs) is paramount to mitigating the risk of vector exposure for animal and human populations living in this area. Finally, the prevention of CD may not be restricted to the control of triatomine vectors by health services. It is necessary to implement educational measures to improve the awareness of the local population regarding the importance of managing the peridomicile environment and avoiding the presence of shelters for these vectors.
