# Supplementary material for: Triatomine vectors of Trypanosoma cruzi in an endemic area for Chagas disease in Northeast Brazil
Source: Rev Soc Bras Med Trop. 2024 Feb 5;57:e00700-2023. doi: 10.1590/0037-8682-0413-2024 (PMC10852468; doi:10.1590/0037-8682-0413-2024)
Supplement: Supplementary file 1 [file 1678-9849-rsbmt-57-e00700-2023-supp1.pdf]

SUPPLEMENTARY TABLE 1: Total number of triatomine species collected and examined in each municipality during the whole study period.

Municipalities	Collected		Total	Examined		Total
	Intra[1]	Peri[2]		Intra	Peri	
Agrestina	54	1	55	54	1	55
Alagoinha	144	15	159	144	15	159
Altinho	32	0	32	32	0	32
Barra de Guabiraba	4	0	4	4	0	4
Belo Jardim	218	23	241	217	22	239
Bezerros	37	3	40	37	3	40
Bonito	15	2	17	15	2	17
Brejo da Madre de Deus	346	4	350	346	4	350
Cachoeirinha	68	11	79	68	11	79
Camocim de São Félix	11	0	11	11	0	11
Caruaru	2,132	142	2,274	2,119	141	2,260
Cupira	65	15	80	65	15	80
Frei Miguelinho	285	10	295	285	10	295
Gravatá	121	0	121	121	0	121
Ibirajuba	36	0	36	36	0	36
Jataúba	22	0	22	22	0	22
Jurema	10	0	10	10	0	10
Panelas	41	1	42	41	1	42
Pesqueira	267	4	271	267	4	271
Poções	37	0	37	37	0	37
Riacho das Almas	15	0	15	15	0	15
Sairé	20	5	25	20	5	25
Sanharó	43	0	43	43	0	43
Santa Cruz do Capibaribe	1,748	213	1,961	1,747	213	1,960
Santa Maria do Cambucá	33	2	35	33	2	35
São Bento do Una	680	6	686	680	6	686
São Caetano	66	0	66	66	0	66
São Joaquim do Monte	3	0	3	3	0	3
Tacaimbó	23	3	26	23	3	26
Taquaritinga do Norte	23	0	23	23	0	23
Toritama	94	5	99	94	5	99
Vertentes	99	0	99	99	0	99
**Total**	**6,792**	**465**	**7,257**	**6,777**	**463**	**7,240**

1: intradomicile; **2:** peridomicile.
